# Analytical performance of the point-of-care *BIOSYNEX COVID-19 Ag BSS* for the detection of SARS‐CoV‐2 nucleocapsid protein in nasopharyngeal swabs: a prospective field evaluation during the COVID-19 third wave in France

**DOI:** 10.1007/s15010-021-01723-5

**Published:** 2021-10-24

**Authors:** Frédéric Fitoussi, Serge Tonen-Wolyec, Natalio Awaida, Raphaël Dupont, Laurent Bélec

**Affiliations:** 1 Laboratoire d’Analyses Médicales, Centre Cardiologique du Nord - CCN, Saint-Denis, France; 2 Ecole Doctorale d’Infectiologie Tropicale, Franceville, Gabon; 3 Faculty of Medicine and Pharmacy, University of Kisangani, Kisangani, Democratic Republic of the Congo; 4 Laboratoire Paris XV, Paris, France; 5 Laboratoire de Virologie, Hôpital Européen Georges Pompidou, Assistance Publique-Hôpitaux de Paris, 20 rue Leblanc, 75015 Paris, France; 6 Sorbonne Paris Cité, Université de Paris, Paris, France

**Keywords:** SARS-CoV-2, COVID-19, Rapid diagnostic test, Lateral flow assay, Antigen, N-nucleocapsid protein, France

## Abstract

**Background:**

The accuracy and reliability of rapid diagnostic tests are critical for monitoring and diagnosing SARS-CoV-2 infection in the general population. This study aimed to evaluate the analytical performance of the *BIOSYNEX COVID-19 Ag BSS* (Biosynex Swiss SA, Fribourg, Switzerland) antigen rapid diagnostic test (*BIOSYNEX* Ag-RDT), which targets the SARS-CoV-2 N-nucleocapsid protein for the diagnosis of COVID-19. The Ag-RDT was compared with a real-time RT-PCR (rtRT-PCR) as gold standard for performance measurement.

**Methods:**

Two nasopharyngeal flocked swabs were prospectively collected simultaneously in March and April 2021 from 967 individuals aged ≥ 18 years tested for SARS-CoV-2 in two private laboratories, Paris, France.

**Results:**

Overall, the Ag-RDT demonstrated high sensitivity, specificity, positive predictive value (PPV), and negative predictive value (NPV) of 81.8%, 99.6%, 96.6%, and 97.5%, respectively. The agreement (97.0%), reliability assessed using Cohen’s κ-coefficient (0.87), and accuracy evaluated using Youden index (J) (81.6%) in detecting SARS-CoV-2 were high. The analytical performance of the Ag-RDT remained high when there was significant viral shedding (*i.e.*, N gene C_t_ values ≤ 33 on reference RT-PCR). The sensitivity was only 55.2% in case of low or very low viral excretion (C_t_ > 33).

**Conclusions:**

The *BIOSYNEX* Ag-RDT is a promising, potentially simple diagnostic tool, especially in symptomatic COVID-19 patients with substantial viral excretion in the nasopharynx.

**Supplementary Information:**

The online version contains supplementary material available at 10.1007/s15010-021-01723-5.

## Introduction

The 2019 coronavirus pandemic (COVID-19) continues to spread worldwide. The effective isolation and early treatment of patients infected by the severe acute respiratory syndrome coronavirus 2 (SARS-CoV-2) require rapid, accurate, and straightforward diagnostic tools.

While currently recommended nucleic acid amplification tests (NAAT), such as real-time reverse transcription-polymerase chain reaction (rtRT-PCR) assays, remain the gold standard cornerstone for the diagnosis of SARS‐CoV‐2 infection [1, 2], viral antigens can be detected using immunological methods [2–4]. Indeed, conducting rtRT-PCR is expensive, time-consuming, and requires special equipment and qualified operators. Point-of-care antigen-detecting rapid diagnostic tests (Ag-RDT) constitute simple and less expensive alternative tests [3]. Ag-RDT relies on direct detection of SARS-CoV-2 viral proteins in nasal swabs and other respiratory secretions. The N-nucleocapsid protein is frequently targeted because of its relative abundance and conserved structure, or other viral proteins such as the spike protein [4]. Most Ag-RDTs rely on sandwich catching using anti-SARS-CoV-2 monoclonal antibodies to detect viral antigens in the simple-to-use lateral flow immunoassay format allowing results in < 30 min. However, significant variability has been reported about their diagnostic performance and a lack of external validation for many available tests, which still require clinical validation [5–9].

Our study aimed to evaluate the qualitative membrane-based immunochromatographic *BIOSYNEX COVID-19 Ag BSS* Ag-RDT (Biosynex Swiss SA, Freiburg, Switzerland; reference SW40006; abbreviated by *BIOSYNEX* Ag-RDT) using monoclonal antibodies detecting SARS-CoV-2 N-nucleocapsid protein to diagnose COVID‐19 from prospectively collected nasopharyngeal secretion samples in adults living in the Paris region throughout the third wave of the COVID-19 epidemic in France.

## Materials and methods

### Rapid antigen test

The *BIOSYNEX* Ag-RDT consists of a reaction membrane and three buffers (sample, reagent, and absorbent). The reagent buffer contains colloidal gold particles conjugated with monoclonal antibodies directed against the N protein of SARS-CoV-2. Secondary antibodies against the N protein are fixed on the reaction membrane. The manufacturer’s instructions were followed to conduct the test by mixing nasopharyngeal secretions with 300 µl of dilution buffer in a tube. After 1 min, four drops were added to the well on the cassette.

If SARS-CoV-2 antigens are present in the sample, the complexes between the anti-SARS-CoV-2 conjugate and the virus are captured by anti-SARS-CoV-2 monoclonal antibodies specific to the test line area (T). The lack of the T line indicates that the result is negative. A red line appears in the control line area (C) to serve as a procedural control, indicating that the correct sample volume has been added and the membrane has played its role. Reading is carried out after 15 min.

### Study population and procedures

During the third wave of the COVID-19 epidemic (March and April 2021), two sites had been used to consecutively collect paired nasopharyngeal swabs. Site A was the *Centre Cardiologique du Nord*, Saint-Denis, France. Site B was the *Laboratoire Paris XV*, Paris, France. Participants aged ≥ 18 years and those consenting to undergo two nasopharyngeal swabs for rtRT-PCR and Ag-RDT were included. All participants were given a questionnaire that recorded demographic information (sex and age), reasons for testing, and current and past 14-day symptoms in symptomatic patients. Suggestive symptoms of COVID-19 were headache, fatigue, fever, or upper or lower respiratory symptoms. Asymptomatic individuals were defined as those not reporting any of these symptoms. At both sites, a health care professional first collected nasopharyngeal secretions in one nostril, using the swab provided in the *BIOSYNEX* Ag-RDT. A second nasopharyngeal swab in the other nostril served as specimen for the rtRT-PCR. The COVID-19 antigen rapid test was performed immediately on-site using the Ag-RDT following the manufacturer’s instructions. The other nasopharyngeal swabs were stored in physiological saline (NaCl 0.9%) (1000 μL) at + 4 °C and analyzed within 24–48 h by the reference rtRT-PCR.

### Molecular detection of SARS-CoV-2

The multiplex real-time PCR Novel Coronavirus (2019-nCoV) Real-Time Multiplex RT-PCR Kit (Detection for 3 Genes) (Liferiver & Shanghai ZJ Bio-Tech Co., Ltd, Shanghai, China) was the reference multiplex molecular detection of SARS-CoV-2 RNA. Individual cycle threshold (C_t_) values for each target gene (E, N and RdRP). According to manufacturer’s recommendations, samples with C_t_ values ≤ 41 for three or two gene targets were considered as positive; those with C_t_ values ≤ 41 for only one gene target were possibly positive; samples with C_t_ value > 41 for the three gene targets were negative.

The C_t_ values of the N gene in the RT-PCR reference were chosen for stratification of viral load in clinical samples because the Ag-RDT detects the SARS-CoV-2 N-nucleocapsid protein.

### Statistical analyses

Collected data were analyzed using IBM® SPSS® Statistics 20 software (IBM, SPSS Inc, Armonk, New York, USA). Results of quantitative variables were expressed as medians; however, the proportion with their 95% confidence interval (CI) assessed according to the Wilson score bounds were estimated for categorical variables [10]. Comparisons were carried out using Pearson’s Chi square test or Fisher’s exact test based on validity conditions. The PPV and NPV were calculated according to Bayes’ formulas, taking into account the officially reported prevalence of SARS-CoV-2 RNA positivity in symptomatic patients in the Paris region on 12th April 2021, e.g., around the peak of the third wave epidemic in France (Santé publique France 2021; https://www.santepubliquefrance.fr/).

### Ethics statement

The purpose of the study was to clinically evaluate the continuous quality improvement program and performance evaluation of COVID-19 management measures following the National Medical-Biological Laboratory Accreditation [11]. The data set was anonymous and contained no identifiable personal health information.

## Results

Paired swab samples were obtained from 967 participants, including 741 from site A and 226 from site B (Table 1). Participants ranged in age from 18 to 95 (median = 34 years). The main reasons for testing were air travel (35.6%), contact-case exposure of an individual infected with SARS-CoV-2 (35.1%), suspected COVID-19 (*n* = 212, 21.9%), preoperative assessment (4.4%), and control of SARS-CoV-2 infection in the previous 30 days (3.0%). The majority (722/967, 74.7%) of included persons were asymptomatic, while a minority (245/967, 25.3%) reported at least one COVID-19-related symptom [including 212 suspected COVID-19 cases, 29 (8.5%) contact cases, 3 (0.9%) travelers, and 1 (3.0%) patient with a recent history of COVID-19]. The median symptom duration before sampling was four days (range, 0–20 days). All comparisons between positive and negative Ag-RDT and rtRT-PCR testing results for both sites and all other variables did not achieve statistical significance (not shown).

**Table 1:** Characteristics of persons providing paired upper respiratory swab (*n* = 967) for real-time reverse transcription-polymerase chain reaction (rtRT-PCR) testing and *BIOSYNEX COVID-19 Ag BSS* rapid diagnostic testing for SARS-CoV-2 at two private laboratory sites, by test results, Paris, France, spring 2021

Characteristics	To number of persons (column %)	Number of persons (row%)			
		rtRT-PCR negative	rtRT-PCR positive	Antigen test negative	Antigen test positive
Total	967 (100)	819 (84.7)	148 (15.3)	844 (87.3)	123 (12.7)
Testing site					
A	741 (74.6)	633 (85.4)	108 (14.6)	651 (87.9)	90 (12.1)
B	226 (25.4)	186 (82.4)	40 (17.6)	193 (85.4)	33 (14.6)
Sex					
Female	498 (51.5)	429 (86.2)	69 (13.8)	438 (88.0)	60 (12.0)
Male	469 (48.5)	390 (83.2)	79 (16.8)	406 (86.6)	63 (13.4)
Age group, years					
18–49	740 (76.4)	629 (85.0)	111 (15.0)	647 (87.4)	93 (12.6)
50–64	157 (16.3)	129 (82.2)	28 (17.8)	133 (84.7)	24 (15.3)
≥ 65	70 (7.3)	61 (87.1)	9 (12.9)	64 (91.4)	6 (8.6)
Median age (range)	34 (18–83)	34 (18–83)	32 (18–82)	34 (18–83)	37 (18–82)
Any symptoms					
≥ 1	245 (25.3)	165 (67.4)	80 (32.6)	168 (68.6)	77 (31.4)
None	722 (74.7)	654 (90.6)	68 (9.4)	676 (93.6)	46 (6.4)
Days from onset of symptoms					
0–3	107 (43.3)	38 (35.5)	69 (64.5)	41(38.3)	66 (61.7)
4–7	122 (49.8)	48 (39.4)	74 (60.6)	69 (56.6)	53 (43.4)
> 7	16 (6.9)	11 (68.7)	5 (31.3)	12 (75.0)	4 (25.0)
Median (range)	4 (0–20)	4 (0–20)	3 (0–15)	4 (0–20)	3 (0–10)
Air travel intention					
Yes	344 (35.6)	330 (95.9)	14 (4.1)	337 (98.0)	7 (2.0)
No	623 (64.4)	489 (78.5)	134 (21.5)	507 (81.4)	116 (18.6)
Contact case (exposure to a diagnosed COVID-19 case)					
Yes	340 (35.1)	282 (82.9)	58 (17.1)	293 (86.2)	47 (13.8)
No/unknown	627 (64.9)	537 (85.7)	90 (14.3)	551 (87.9)	76 (12.1)
Suspected COVID-19					
Yes (≥ 1 COVID-19 symptoms	212 (21.9)	149 (70.3)	63 (29.7)	151 (71.2)	61 (28.8)
No	755 (78.1)	670 (88.8)	85 (11.2)	693 (91.8)	62 (8.2)
Preoperative assessment					
Yes	42 (4.4)	38 (90.5)	4 (9.5)	40 (95.2)	2 (2.8)
No	925 (95.6)	781 (84.4)	144 (15.6)	804 (86.9)	121 (13.1)
Control of SARS-CoV-2 positive test results in past 30 days					
Yes	29 (3.0)	20 (68.9)	9 (31.1)	21 (72.4)	8 (27.6)
No/unknown	938 (97.0)	799 (85.2)	139 (14.8)	823 (87.7)	115 (12.3)

Among the 148 positive samples using the gold standard rtRT-PCR, 146 were positive for the three gene targets, and two were positive for only E and N genes. The mean ± SD of the C_t_ values were 26.1 ± 4.4 arbitrary units (a.u.) for the E gene, 26.5 ± 5.0 a.u. for the RdRP gene, and 26.9 ± 5.1 a.u. for the N gene.

The vast majority (114/123, 92.7%) of positive results were visible in the window of the cassette of the Ag-RDT within the first 5 min. Table 2 shows the test results and primary performance characteristics of the *BIOSYNEX* Ag-RDT compared with the reference rtRT‐PCR in the study population according to COVID-19-compatible symptoms. Using rtRT-PCR as the standard, three false-positive *BIOSYNEX* Ag-RDT results occurred among specimens from asymptomatic individuals (*n* = 2) or symptomatic patients (*n* = 1). Of the 148 rtRT-PCR positive results, 27 (18.2%) were false-negative *BIOSYNEX* Ag-RDT (23 specimens from asymptomatic persons and 4 specimens from symptomatic patients). Overall, the *BIOSYNEX* Ag-RDT showed high sensitivity (81.8%), specificity (99.6%), PPV (96.6%), and NPV (97.5%). Among symptomatic patients, sensitivity was 95.0%, specificity was 99.4%, PPV was 95.6%, and NPV was 96.3% (Table 2). Within 7 days from symptom beginning, the *BIOSYNEX* Ag-RDT showed a sensitivity of 96.6%, a specificity of 99.4%, whereas the PPV and NPV were 95.7% and 99.4%, respectively.

**Table 2:** Test results and performances characteristics of the *BIOSYNEX COVID-19 Ag BSS* rapid diagnostic test compared with real-time reverse transcription-polymerase chain reaction (rtRT-PCR) for SARS-CoV-2 testing among asymptomatic and symptomatic persons at two private laboratory sites, by test results, Paris, France, spring 2021

Results and performances	rtRT-PCR (number of test, %)		Total
	Positive	Negative	
BIOSYNEX COVID-19 Ag BSS results			
All participants (*n* = 967)			
Positive	121 (12.5)	3 (0.3)	124 (12.8)
Negative	27 (2.8)	816 (84.4)	843 (87.2)
Total	148 (21.2)	819 (78.8)	967 (100)
Asymptomatic (*n* = 722)			
Positive	45 (4.2)	2 (0.3)	47 (6.5)
Negative	23 (5.2)	652 (90.3)	675 (95.5)
Total	68 (9.4)	654 (90.6)	722 (100)
Symptomatic (≥ 1 symptom) (*n* = 245)			
Positive	76 (31.0)	1 (0.4)	77 (31.4)
Negative	4 (1.6)	164 (67.0)	168 (68.6)
Total	80 (32.6)	165 (67.4)	245 (100)
Symptomatic (≤ 7 days from symptom onset) (*n* = 229)			
Positive	72 (31.4)	1 (0.5)	73 (31.9)
Negative	3 (1.3)	153 (66.8)	156 (68.1)
Total	75 (32.7)	154 (67.3)	229 (100)
*BIOSYNEX COVID-19 Ag BSS* performances (%, 95%CI)			
All participants			
Sensitivity		81.8 (79.2–84.1)	
Specificity		99.6 (98.9–99.8)	
PPV^£^		96.6 (95.3–97.6)	
NPV^£^		97.5 (96.3–98.3)	
Asymptomatic			
Sensitivity		79.4 (76.3–82.2)	
Specificity		99.7 (98.9–99.9)	
PPV		97.3 (95.8–98.2)	
NPV		97.2 (95.7–98.2)	
Symptomatic			
Sensitivity		95.6 (92.2–97.5)	
Specificity		99.3 (97.2–99.8)	
PPV		95.6 (92.2–97.5)	
NPV		99.3 (97.2–99.8)	
Symptomatic (≤ 7 days from onset)			
Sensitivity		96.0 (92.6–97.9)	
Specificity		99.4 (97.3–99.9)	
PPV		95.7 (92.2–97.7)	
NPV		99.4 (97.3–99.9)	

*CI* confidence interval; *NVP* negative predictive value; *PPV* positive predictive value

^£^PPV and NPV were calculated according to the Bayes’s formulae, by taking into account the official reported prevalence of SARS-CoV-2-RNA positivity in COVID-19-suspected patients in Paris’s area, France, of 12.2% on 12th April 2021 [Santé publique France 2021; https://www.santepubliquefrance.fr/]

The Table 3 shows the analytical results based on the level of viral excretion assessed by the N gene C_t_ values using the reference rtRT‐PCR. Overall, the *BIOSYNEX* Ag-RDT showed high agreement (97.0%), reliability using Cohen’s κ coefficient (0.87), and accuracy using Youden’s J index (81.6%) to detect SARS-CoV-2.

**Table 3:** Analytical performances of the *BIOSYNEX COVID-19 Ag BSS* rapid diagnostic test for the qualitative detection of the N protein of SARS-CoV-2 using 967 prospectively collected nasopharyngeal swab samples by reference rtRT-PCR^**#**^, according to their N gene C_t_ values

N gene C_t_ (median; range)			*N*	BIOSYNEX COVID-19 Ag BSS^§^								
				FN (*n*)	TP (*n*)	Sensitivity^a^ (% [95% CI])^µ^	Specificity^a^ (% [95% CI])	Agreement^b^	Concordance^c^	Youden’s J index^d^	PPV^e^% [95% CI])	NPV^e^ (% [95% CI])
Detectable N gene C_t_^£^ by rtRT-PCR^#^	≤ 20	17.9 (13.9–20.0)	35	0	35	100 (99.6–100)	99.6 (98.9–99.8)	99.6 (98.9–99.8)	0.95 (0.93–0.96)	99.6 (98.9–99.8))	97.2 (95.9–98.1)	100 (99.6–100)
	21–33	27.2 (20.1–33.0)	84	14	70	83.3 (80.7–85.6)	99.6 (98.9–99.8)	98.1 (97.0–98.8)	0.65 (0.62–0.68)	82.9 (80.3–85.2)	96.7 (95.3–97.7)	97.7 (96.5–98.5)
	> 33–41	35.9 (34.0–39.2)	29	13	16	55.2 (51.8–58.5)	99.6 (98.9–99.8)	98.1 (96.9–98.8)	0.65 (0.62–0.68)	54.8 (51.4–58.1)	95.0 (93.3–96.3)	94.1 (92.3–95.5)
	All positive C_t_ values	26.9 (13.9–39.2)	148	27	121	81.8 (79.2–84.1)	99.6 (98.9–99.8)	96.9 (95.9–97.8)	0.87 (0.85–0.89)	81.4 (78.8–83.7)	96.6 (95.3–97.6)	97.5 (96.3–98.3)

*C*_*t*_ cycle threshold; *FN* false negative; *NPV* negative predictive value; *PPV* positive predictive value; *rtRT-PCR* real-time reverse transcription-polymerase chain reaction; *TP* True positive

^§^Paired nasopharyngeal samples in each nostril were collected with a flocked swab for each volunteer patients by trained healthcare personnel (nurses, doctors or biologists). The collection of the two simultaneous samples was always carried out by the same operator. Molecular testing as well COVID-19 antigen detection was carried out on fresh samples.

^a^The results of SARS-CoV-2 RNA detection using the multiplex rtRT-PCR were used as the reference standard to estimate the sensitivity and specificity of the study Ag-RDT, with corresponding 95% CI.

^b^Agreement = TP + TN/TP + FP + TN + FN, expressed in percentage.

^c^The Cohen’s κ coefficient calculation was used to estimate the concordance [43] and interpreted according the Landis and Koch scale [44], as follows: < 0 as indicating no agreement, 0–0.20 as slight, 0.21–0.40 as fair, 0.41–0.60 as moderate, 0.61–0.80 as substantial, and 0.81–1 as almost perfect concordance.

^d^The accuracy of the test *BIOSYNEX COVID-19 Ag BSS* to correctly diagnose SARS-CoV-2 infection was estimated by Youden’s J index (J = sensitivity + specificity − 1) [45].

^e^PPV and NPV were calculated according to the Bayes’s formulae, by taking into account the official reported prevalence of SARS-CoV-2-RNA positivity in COVID-19-suspected patients in Paris’s area, France, of 12.2% on 12th April 2021 [Santé publique France 2021; https://www.santepubliquefrance.fr/].

^µ^95% confidence intervals in brackets were calculated using the Wilson score bounds.

^£^The C_t_ values of N gene detection by the reference Liferiver rtRT-PCR were used to classify nasopharyngeal samples according to their level of SARS-CoV-2 RNA excretion; C_t_ of 20 and 33 were taken as thresholds of very high and high SARS-CoV-2 RNA excretion, respectively, as previously stated [36, 39, 40, 41] ; viral loads with C_t_ > 33 are considered low and correspond to moderate or very low viral excretion [36, 39–41]. Conversely, samples with C_t_ ≤ 33 have a significant SARS-CoV-2 viral load, as in individuals symptomatic for COVID-19 or contagious. C_t_ values ≤ 20 indicate very high viral shedding [39–41]

^**#**^The CE IVD-marked Novel Coronavirus (2019-nCoV) Real-Time Multiplex RT-PCR Kit (Detection for 3 Genes) (Liferiver & Shanghai ZJ Bio-Tech Co., Ltd, Shanghai, China) constituted the reference multiplex rtRT-PCR for SARS-CoV-2 RNA detection. This assay can detect three coronavirus target genes simultaneously, including the SARS-like (SARS-CoV-2, SARS-CoV, bat SARS-like coronavirus) conserved region of envelope protein gene (E), RNA-dependent RNA polymerase gene (ORF1ab of RdRP gene), and nucleocapsid protein gene (N), using reverse transcription. Nucleic acid extraction was performed from 300 μL elution volume of a nasopharyngeal flocked swab sample, using an EX3600 extractor (Liferiver & Shanghai ZJ Bio-Tech Co.), according to the manufacturer’s instructions, and finally eluted in 50 μL (final volume). SARS-CoV-2 was detected in 5 μL of extracted RNA. Real-time PCR was conducted using CFX96™ Real-Time PCR Detection System (Bio-Rad Laboratories, Hercules, CA, USA) according to the manufacturer’s instructions. The experiment and analysis of the results were performed according to the manufacturer’s protocol.

In case of high or very high viral loads (C_t_ ≤ 33), the *BIOSYNEX* Ag-RDT had a good analytical performance (sensitivities between 83.3% and 100.0%, specificities of 99.8%, PPV between 98.3% and 98.6%, and NPV between 97.7% and 100.0%). In case of low or very low viral loads (C_t_ > 33), the sensitivity of the *BIOSYNEX* Ag-RDT had reduced analytical performance (sensitivity of only 55.2%), while its specificity remained high (98.8%). Similar observations were made when the C_t_ values of the E or ORF1ab gene targets were chosen for stratification of viral load in clinical samples (data not shown).

Finally, the sensitivity of the *BIOSYNEX* Ag-RDT varied among the five participant groups as follows: (i) travel: 50.0% (7/14), (ii) contact-case exposure: 81.0% (47/58), (iii) preoperative assessment: 50.0% (2/4); (iv) suspected COVID − 19: 96.8% (61/63), and (v) control of SARS-CoV-2 positive test results in the last 30 days: 88.9% (8/9).

## Discussion

We evaluated the analytical performance of the novel point-of-care *BIOSYNEX* Ag-RDT compared to multiplex rtRT-PCR as gold standard for detecting SARS-CoV-2 RNA in a real-life setting. In this study, the sensitivity of the *BIOSYNEX* Ag-RDT was lower among specimens from asymptomatic persons (79.4%) than among specimens from symptomatic patients (95.0%). It was high in patients with suspected COVID-19 (96.8%). Specificity (> 99.0%) was high in specimens from both asymptomatic individuals and symptomatic patients. The prevalence of SARS-CoV-2 RNA-positive rt-RT-PCR results in this population was 15.3% overall, 9.4% for asymptomatic individuals, and 32.6% for symptomatic patients. The estimated PPVs and NPVs of the *BIOSYNEX* Ag-RDT were elevated in all groups of participants. However, administering the Ag-RDT in low prevalence settings will likely result in lower predictive values. In the event of significant viral excretion (i.e., N gene C_t_ values below 33 based on reference rtRT-PCR), the *BIOSYNEX* Ag-RDT showed high sensitivity (from 83.3% to 100.0%) and specificity (> 99.0%) for SARS-CoV-2 RNA detection. Concordance, reliability, as well as accuracy were great with the reference assay and PPVs and NPVs above 97.0%. However, the sensitivity of the study Ag-RDT dropped to 55.2% with low or very low viral shedding (C_t_ > 33). Together, these observations demonstrated the high analytical performance of the *BIOSYNEX* Ag-RDT. This performance made it suitable for use as point-of-care Ag-RDT in various hospital and non-hospital settings where a rapid diagnosis of SARS-CoV-2 is necessary. Although less sensitive than RT-PCR, the *BIOSYNEX* Ag-RDT could be beneficial by obtaining quick results, ease of use, and independence from existing laboratory structures. Testing criteria focusing on patients during the early onset of symptoms could further increase its diagnostic value.

The sensitivity of the *BIOSYNEX* Ag-RDT was 81.8% overall, and the positive detection rate was comparable to the rtRT-PCR in the majority (88.2%) of subjects with C_t_ ≤ 33. False-negative test results of 12/14 (85.7%) subjects with significant viral excretion (C_t_ ≤ 33) were asymptomatic, although conflicting evidence exists about the relationship between symptom severity and viral shedding [12]. False-positive test results were rarely observed, providing 99.6%-specificity, exceeding the performance recommended by the World Health Organization (WHO) [13]. False-positive results have been reported as well in other antigen tests [14–16]. False positivity could be associated with high viscosity of tested specimen samples as well as interference with mucosal antibodies [17].

Finally, the *BIOSYNEX* Ag-RDT meets the current WHO criteria which stipulate that Ag-RDTs for SARS-CoV-2 antigen detection must have a sensitivity greater than 80% and a specificity greater than 97% (97%–100%) [13]. Furthermore, analytical performances comparable to those in our study Ag-RDT were previously reported for some Ag-RDTs in lateral flow immunoassay format [7, 9, 14, 18–28], while several studies have reported much lower sensitivity levels contrasting with consistently high specificity [3, 29–34]. In addition, the *BIOSYNEX* Ag-RDT also fulfilled the current recommendations of the French High Authority of Health (*Haute Autorité de santé*, Saint-Denis, France) for a screening Ag-RTD stating that, at minimum, Ag-RDTs would need to correctly identify significant proportions of symptomatic patients (sensitivity ≥ 80%) as well as asymptomatic individuals (sensitivity ≥ 50%) and have high specificity (≥ 90%) [35].

We analyzed our results based on the estimated viral load in SARS-CoV-2 in the samples. There is an ongoing debate about the C_t_ value corresponding to the threshold of infectivity (i.e., patient considered as contagious) [7, 36, 37]. La Scola et al. found that patients with C_t_ values > 33 are not infectious because of the low number of positive cultures [38]. The Centers for Disease Control and Prevention (CDC), Atlanta, USA, propose a C_t_ cut-off value of 33 as a marker for contagiousness [39], and stress that C_t_ values ≤ 20 correspond to very high viral excretion [7, 36, 40, 41]. Our results confirm that the analytical performances of the *BIOSYNEX* Ag-RDT were much better in specimens with a high viral load. These observations demonstrate the capability of the *BIOSYNEX* Ag-RDT as a rapid rule-in test for COVID-19 with samples at high viral load in symptomatic patients, for example, and raise caution about its use as a singular rule-out test, especially in samples with lower viral loads.

Our study has several strengths. All samples were collected from one nasopharynx with flocked swabs, optimal for evaluating Ag-RDT clinical performances in our study. The Ag-RDT and reference rtRT-PCR were carried out in parallel. The study population included various situations outside the hospital setting, with mostly young adults without comorbidities who had typical and mild COVID-19 symptoms when being symptomatic.

The study presents also some limitations. Participants may have inadvertently reported general, non-specific symptoms as COVID-19 compatible symptoms. This investigation evaluated the *BIOSYNEX* Ag-RDT; the results presented here cannot be generalized to other agencies-authorized SARS-CoV-2 antigen tests. Otherwise, the CDC clarified that C_t_ values using the rtRT-PCR platform is not a quantitative measure of viral burden in clinical samples and cannot be used to assess whether a person is infectious [42]. Consequently, our stratification of samples according to C_t_ values of the N gene does not necessarily reflect the actual infectivity of the participants. Finally, higher rate of asymptomatic persons in the study (with lower virus level) could have resulted in decreased sensitivity of the *BIOSYNEX* Ag-RDT.

## Conclusion

The *BIOSYNEX* Ag-RDT demonstrated high specificity and sufficient sensitivity for detecting SARS-CoV-2. Given the simple procedures and short turnaround time for this test, it is a promising option as an alternative diagnostic modality, especially in symptomatic COVID-19 patients. The test may also be used to test asymptomatic individuals suspicious of exposure to SARS-CoV-2 and as part of a population-level mass screening.

## Supplementary Information

Below is the link to the electronic supplementary material.

Supplementary file1 (XLSX 153 KB)

## Data Availability

Study data are available and can be used for academic or research purposes.

## Abbreviations

Ag-RDT: Antigen-detecting rapid diagnostic test
*BIOSYNEX* Ag-RDT: *BIOSYNEX* COVID-19 Ag BSS
CDC: Centers for Disease Control and Prevention
COVID-19: Coronavirus disease 2019
NAAT: Nucleic acid amplification test
rtRT‐PCR: real-time reverse transcription-polymerase chain reaction
SARS-CoV-2: Severe acute respiratory syndrome coronavirus 2
WHO: World Health Organization
